# The Astrocytic S100B Protein with Its Receptor RAGE Is Aberrantly Expressed in SOD1^G93A^ Models, and Its Inhibition Decreases the Expression of Proinflammatory Genes

**DOI:** 10.1155/2017/1626204

**Published:** 2017-06-20

**Authors:** Alessia Serrano, Claudia Donno, Stefano Giannetti, Mina Perić, Pavle Andjus, Nadia D'Ambrosi, Fabrizio Michetti

**Affiliations:** ^1^Institute of Anatomy and Cell Biology, Università Cattolica del Sacro Cuore, Rome, Italy; ^2^Institute of Physiology and Biochemistry, Faculty of Biology, University of Belgrade, Belgrade, Serbia; ^3^Department of Biology, University of Rome “Tor Vergata”, Rome, Italy; ^4^IRCCS San Raffaele Scientific Institute, Università Vita-Salute San Raffaele, Milan, Italy

## Abstract

Neuroinflammation is one of the major players in amyotrophic lateral sclerosis (ALS) pathogenesis, and astrocytes are significantly involved in this process. The astrocytic protein S100B can be released in pathological states activating the receptor for advanced glycation end products (RAGE). Different indications point to an aberrant expression of S100B and RAGE in ALS. In this work, we observed that S100B and RAGE are progressively and selectively upregulated in astrocytes of diseased rats with a tissue-specific timing pattern, correlated to the level of neurodegeneration. The expression of the full-length and soluble RAGE isoforms could also be linked to the degree of tissue damage. The mere presence of mutant SOD1 is able to increase the intracellular levels and release S100B from astrocytes, suggesting the possibility that an increased astrocytic S100B expression might be an early occurring event in the disease. Finally, our findings indicate that the protein may exert a proinflammatory role in ALS, since its inhibition in astrocytes derived from SOD1^G93A^ mice limits the expression of reactivity-linked/proinflammatory genes. Thus, our results propose the S100B-RAGE axis as an effective contributor to the pathogenesis of the disease, suggesting its blockade as a rational target for a therapeutic intervention in ALS.

## 1. Introduction

Neuroinflammation is believed to be one of the major players in amyotrophic lateral sclerosis (ALS) pathogenesis. Although the primary cause of motor neuron degeneration is likely to be intrinsic to motor neurons themselves, glial cells surrounding damaged neurons are at least responsible for the progression of the disease [1–3]. The strict relationship between neuroinflammatory events and ALS phenotype has led to intense investigations aimed at understanding the pathways triggering glial activation and the processes characterizing the consequent response of glial cells [4, 5], with the purpose to find motor neuron-alternative targets to counteract the course of the disease (for review [6]).

The role of astrocytes, hubs of homeostatic control in the physiology of the central nervous system (CNS), in the etiopathogenesis of ALS is accordingly a significant field of study. An increasing reactive astrocytosis is found in affected areas of the nervous system in ALS, with presence of hypertrophic astrocytes surrounding degenerating motor neurons and formation of a glial scar in late phases of the disease [7]. Moreover, astrocytes expressing ALS-linked mutations, as well as those originating from sporadic ALS (sALS) patients, are toxic to motor neurons in culture [8–11]. The cytotoxicity is mediated by released soluble molecules [12, 13], which thorough identification could be of pivotal importance to understand and eventually control the disease progression.

During injury, endogenous alarm signals, known as danger-associated molecular patterns (DAMPs), are released by activated, damaged, or dying cells, including glia and neurons. The recognition of such molecules is allowed by the expression of specific pattern-recognition receptors (for review [14]). S100B, a calcium-binding protein mainly expressed by astrocytes in CNS in healthy conditions, is believed to exert trophic actions when released at low (putatively physiological) concentrations, while at high concentrations it is regarded to behave as a DAMP, participating in the cascade of events leading to cell injury. S100B also shares with this class of molecule features such as the interaction with the receptor for advanced glycation end products (RAGE), the noncanonical secretion modality that bypasses the classical Golgi route, and the ability to stimulate microglial migration (for review [15]). RAGE recruitment may occur in nearby neurons or in astrocytes, respectively, through a paracrine mechanism [16] or an autocrine self-regenerating pathway [17], with a still debated outcome. The overall effect exerted by S100B is also influenced by the existence of different RAGE isoforms: in addition to the full-length receptor (FL-RAGE), there are splicing and proteolytic variants that represent soluble forms (s-RAGE) of the receptor that act as decoy molecules, sequestering the receptor ligands without triggering a response [18].

A dysregulation of the S100B-RAGE system and its implication with pathological events have been observed in several neurodegenerative diseases, as Parkinson's (PD) and Alzheimer's (AD). In AD in particular, RAGE exerts a critical action in A*β* clearance [19], while in PD the ablation of S100B results in neuroprotection, reduced microgliosis and expression of both RAGE and tumor necrosis factor (TNF)*α* [20]. In ALS, different indications point to an aberrant expression of S100B: in patients, S100B is increased in astrocytes in the cortex [21], in astrocytes, and motor neurons in the spinal cord [22] and its levels are also raised in the cerebrospinal fluid (CSF), positively correlating with a worse prognosis of the disease [23]. In the spinal cord of rodent models of ALS, S100B overexpression was documented only in astrocytes [24–26], even if the presence of the protein in other cell types has not been directly investigated. Interestingly, in SOD1^G93A^ rats, the increase of S100B concerns a subpopulation of astrocytes, defined as “aberrant” and characterized by a typical molecular signature (increase in connexin (CX)-43 and decrease of glutamate transporter (GLT)-1) and by an increased toxicity towards motor neurons, mediated by unidentified soluble molecules [26]. In addition, immunohistochemical studies performed in human lumbar spinal cord have demonstrated the presence of RAGE in motor neurons, but not in resting glial cells in control samples, while in ALS tissues, when motor neurons are lost, RAGE appears to be increased in cells with typical morphologies of astrocytes and microglia; this situation is reflected by an overall stability of total RAGE mRNA levels [27]. Nevertheless, in the mouse model of the disease, RAGE staining in the spinal cord is visible only in affected animals (with no indications about its cellular localization) [28].

The present study offers comprehensive information concerning the aberrant expression of the S100B/RAGE axis in SOD1^G93A^ models and assesses the direct influence of S100B on the expression of reactivity-linked/proinflammatory molecules in astrocytes from mutant SOD1 mice.

## 2. Materials and Methods

### 2.1. Animals

Experiments were performed on wild type (WT) and transgenic Sprague-Dawley male rats, carrying human-mutated SOD1^G93A^ (002148-T, NTac: SD-Tg (SOD1^G93A^) L26H; Taconic, Hudson, NY, USA). Transgenic SOD1^G93A^ animals were identified by polymerase chain reaction using tail clip genomic DNA for the presence of transgene [29]. ALS-like disease stages were determined using a combination of body weight measurement and neurological score assessment. Animals were assigned presymptomatic (ps-SOD1^G93A^) at the age of approximately 7 months with no clinical signs of disease and at the peak of body weight-time curve. Disease onset was observed at the age of approximately 8 months, and end-stage animals (es-SOD1^G93A^) were sacrificed when the atrophy of both hind limbs was detected accompanied with a significant loss of body mass. In each experiment, age-matching WT animals were sacrificed in parallel with ps-SOD1^G93A^ and es-SOD1^G93A^ animals. All animals were maintained in a 12 h light/dark cycle and had ad libitum access to food and water. All experiments were performed according to the rules for animal care proposed by the Serbian Laboratory Animal Science Association, a member of the Federation of the European Laboratory Animal Science Associations and approved by the Ethics Committee of the Faculty of Biology, University of Belgrade.

### 2.2. Antibodies

The following primary antibodies were used for immunofluorescence: anti-rabbit S100B (1 : 7500, Novus Biological), anti-mouse S100B (1 : 1000, Sigma Aldrich), anti-mouse RAGE (1 : 200, Millipore), anti-rabbit GFAP (1 : 1000, Dako), anti-mouse GFAP (1 : 1000, Novus Biologicals), anti-mouse NeuN (1 : 500, Millipore), anti-rabbit Iba1 (1 : 200, Wako), anti-mouse CNPase (1 : 500, Novus Biologicals), and anti-rabbit ChAT (1 : 200, Millipore). Secondary fluorescent antibodies were Cy3 Donkey anti-rabbit (1 : 200), Alexa-Fluor 488 Donkey anti-rabbit (1 : 200), Cy3 Donkey anti-mouse (1 : 200), and Alexa Fluor 488 Donkey anti-mouse (1 : 200) from Jackson ImmunoResearch Laboratories. To-Pro-3 (1 : 10,000, Thermo Fisher Scientific) was used to stain nuclei. The primary antibodies used for Western blotting were anti-S100B (1 : 1000, Novus Biologicals), anti-rabbit RAGE (1 : 1000, Thermo Scientific), anti-mouse RAGE (1 : 1000, Millipore), anti-GAPDH (1 : 10,000, Millipore), anti-SOD1 (1 : 1000, Enzo Life Sciences), and anti-GFAP (1 : 5000, Novus biologicals). Anti-rabbit and anti-mouse IgG peroxidase-conjugated secondary antibodies (1 : 2500) were from Bio-Rad Laboratories.

### 2.3. Immunofluorescence Microscopy

The ps-SOD1^G93A^ (*n* = 3), es-SOD1^G93A^ (*n* = 3), and WT (*n* = 6) rats were anaesthetized by intraperitoneal injection of xylazine (0.12 ml/100 g b.w.) and ketamine (0.06 ml/100 g b.w.) and transcardially perfused with saline solution and 4% paraformaldehyde (PFA) in 0.1 M phosphate buffer (PB). Tissue samples were postfixed overnight in 4% PFA and then cryoprotected in increasing concentrations of sucrose (10%, 20%, and 30%) in 0.1 M PB at 4°C. Prior to cryosection, cervical and lumbar spinal cords were frozen at −80°C and horizontal sections of 30 *μ*m thickness were cut on the cryotome (CM 1850, Leica, Germany). Double immunofluorescence analysis was performed in free floating according to the following procedure: sections were washed in PBS and blocked in PBS containing 10% normal donkey serum and 0.3% Triton X-100 for 1 hour at room temperature. Spinal cord sections were incubated with the appropriate antibodies in PBS, 2% normal donkey serum, and 0.3% Triton X-100 over night at 4°C. Slides were washed with PBS and incubated with appropriate fluorescent-conjugated secondary antibodies in PBS, 1% normal donkey serum, and 0.3% Triton X-100 for 3 hours at room temperature. After a PBS wash, sections were incubated with the nuclear marker To-Pro-3 for 5 minutes at room temperature. The slides were coverslipped with Vectashield mounting medium (Vector Laboratories). Immunofluorescences with either anti-S100B (Sigma), anti-RAGE (Millipore), or anti-CNPase were performed without Triton X-100. Immunofluorescence was analyzed by means of a confocal laser scanning microscope (LSM 510 META, Zeiss) equipped with three lasers: Argon/2, HeNe543, and HeNe633. The brightness and contrast of the digital images were adjusted using the LSM Image Browser software (Zeiss).

### 2.4. Nissl Staining and Motor Neuron Count

The tissue was processed as described in the previous paragraph, and spinal cord sections (*n* = 3 per condition) were randomly selected and stained with 1% cresyl violet. Stained sections were dehydrated gradually in 50–100% alcohol, cleared in xylene, and coverslipped with Eukitt (Sigma-Aldrich). The whole ventral horn of the spinal cord was photographed at ×10 magnification with Zeiss Axiophot microscope. Large neurons, with cell bodies ≥200 *μ*m and a definable cytoplasm with a nucleus and nucleolus [30], were then counted.

### 2.5. Cell Cultures, Transfection, and Silencing

C6 rat astrocytoma cells were grown in Dulbecco's modified Eagle's medium (DMEM, Euroclone) supplemented with 10% fetal bovine serum (Gibco) at 37°C in an atmosphere of 5% CO_2_ in air. To perform transfection, C6 cells were plated at 70% confluency and, 24 h later, transfected with pCMV, pCMV-SOD1^wt^, or pCMV-SOD1^G93A^ using Metafectene Pro reagent (Biontex) according to the manufacturer's instruction. Cells were harvested 24 h after transfection. Primary astrocytes were obtained according to the protocol of Mecha et al. [31]. For the S100B silencing, primary astrocytes were plated and transfected with 100 ng/ml of scramble siRNA (si-Scr # 4611) or with 50 ng/ml each of a pair of S100B siRNAs (si-S100B #64409 and #287335) (Ambion, Thermo Fisher Scientific) using Metafectene Pro reagent (Biontex) according to the manufacturer's instruction. To obtain an improved silencing efficiency, both negative controls and si-s100B-treated cells were transfected twice, at 24 and 48 hours, and harvested at 72 hours post the first transfection.

### 2.6. Protein Extraction, SDS-PAGE, and Western Blotting

Protein lysates from cervical and lumbar spinal cord segments were obtained according to the following procedure: tissues were crushed with a potter in RIPA buffer (PBS, 1% Nonidet P-40, 0.5% sodium deoxycholate, 0.1% SDS) containing a protease inhibitor cocktail (Cell Signaling). After an incubation of 30 minutes in ice, the lysates were centrifuged for 20 min at 14.000*g* at 4°C. Supernatants were collected and assayed for protein quantification with the Bradford detection kit (Bio-Rad Laboratories). To isolate total-protein extracts from cellular cultures, cells were harvested in ice-cold RIPA buffer added with protease inhibitor cocktail (Cell Signaling). Lysates were kept on ice for 30 min and then centrifuged for 10 min at 14.000*g* at 4°C. Supernatants were collected and assayed for protein quantification with the BCA protein assay (Thermo Fisher Scientific). Protein samples were separated by SDS-PAGE and transferred onto PVDF membranes (GE Healthcare). Membranes were blocked for 1 h in Tris-buffered saline solution with 0.1% Tween-20 (TBS-T) containing 5% nonfat dry milk and then incubated for 2 h at room temperature or overnight at 4°C with indicated primary antibodies diluted in TBS-T containing 2% nonfat dry milk. After rinsing with TBS-T solution, membranes were incubated for 1 h with the appropriated peroxidase-conjugated secondary antibody diluted in TBS-T containing 1% nonfat dry milk, then washed and developed using the ECL chemiluminescence detection system (Roche). Densitometric analyses were performed using ImageJ software program (National Institutes of Health).

### 2.7. S100B Analysis by ELISA

Culture medium (100 *μ*l) from C6 cultures transfected with pCMV, pCMV-SOD1^wt^, or pCMV-SOD1^G93A^ was collected and measured for S100B content using S100B human ELISA kit (BioVendor) following the manufacturer's instructions. The HRP-generated signal was detected by a microplate reader at 450 nm, and the OD values were converted in S100B concentration using a standard curve.

### 2.8. Semiquantitative PCR and Real-Time qPCR

For quantitative Real-Time PCR and semiquantitative PCR, RNAs were isolated using TRIzol (Thermo Fisher Scientific) for tissue extraction and RNeasy kit (Qiagen) for the cell cultures. RNAs were quantified and reverse-transcribed with random primers by GoScript Reverse Transcription System (Promega). qRT-PCR was performed with GoTaq qPCR Green Master Mix (Promega) according to the manufacturer's instruction. The primers used were the following:

Fw CCL 65′-AGC CGG GCA TCA TCT TTA TCA G-3′

Rv CCL 65′-AGA TCT TGG GCC TTG CTT CAG-3′

Fw CXCL 105′-ATG ACG GGC CAG TGA GAA TG-3′

Rv CXCL 105′-TCG TGG CAA TGA TCT CAA CAC-3′

Fw GAPDH5′-AAG GGC TCA TGA CCA CAG TC-3′

Rv GAPDH5′-GGG CCA TCC ACA GTC TTC T-3′

Fw GFAP5′-GAT CCG AGG GGG CAA AAG C-3′

Rv GFAP5′-GGC AGG GCT CCA TTT TCA ATC-3′

Fw TNF*α*5′-CAG ACC CTC ACA CTC AGA TCA-3′

Rv TNF*α*5′-CTT GGT TTG CTA CGA CG-3′

Fw S100B5′-TGG AGG AAA TCA AAG AGC-3′

Rv S100B5′-CAG CGT CTC CAT CAC TTT GT-3′

Semiquantitative PCR was performed with BioMix Red (Bioline) according to the manufacturer's instruction, and the primers used were the following:

Fw ACT5′-GACCCAGATCATGTTTGAGACCT-3′

Rv ACT5′-ACC AGA GGC ATA CAG GGA CA-3′

Fw RAGE 5′-CAT CAG GGT CAC AGA AAC CG-3′

Rv FL-RAGE5′-CCT TCC TCT CCT CAC GCC T-3′

Rv sRAGE5′-TCC AGT CCC TCA CCT TCA GC-3′

Primers were designed to discriminate between the FL and the s-RAGE isoforms, since the Rv sRAGE primer pairs with a sequence on intron 9, which is absent in the FL-RAGE mRNA. A mix containing the Fw and both Rv oligos was then amplified for 30 cycles. The two isoforms could then be resolved by their length. Densitometric analyses were performed using ImageJ software program, and the ratio between FL-RAGE and sRAGE was reported.

### 2.9. Statistical Analysis

Data are presented as mean ± s.d. Analysis was performed with the statistical software package MedCalc (Medcalc Software, Mariakerke) using ANOVA. Statistical differences between groups were verified by Student's *t*-test. ^∗^*P* < 0.05 was considered significant.

## 3. Results

### 3.1. The Levels of S100B in the Lumbar Spinal Cord Increase Selectively in Astrocytes during the Course of the Disease

In the first instance, we evaluated the extent of motor neuron degeneration in affected tissues of presymptomatic (ps-SOD1^G93A^) and end-stage (es-SOD1^G93A^) rats (Supplementary Figure S1A and C available online at https://doi.org/10.1155/2017/1626204). Although a certain extent of motor neuron degeneration is already present in the lumbar spinal cord of our ps-SOD1^G93A^ rats (25% compared to WT), these observations are in line with previous data [32, 33], also reporting that the onset of muscle weakness occurs when the loss of motor neurons from the spinal cord reaches a threshold of about 50% [33]. A systematic analysis on the presence and localization of S100B and RAGE in the lumbar and cervical spinal cord from ps- and es-SOD1^G93A^ rats with respect to control animals was then performed.

**Figure 1:**
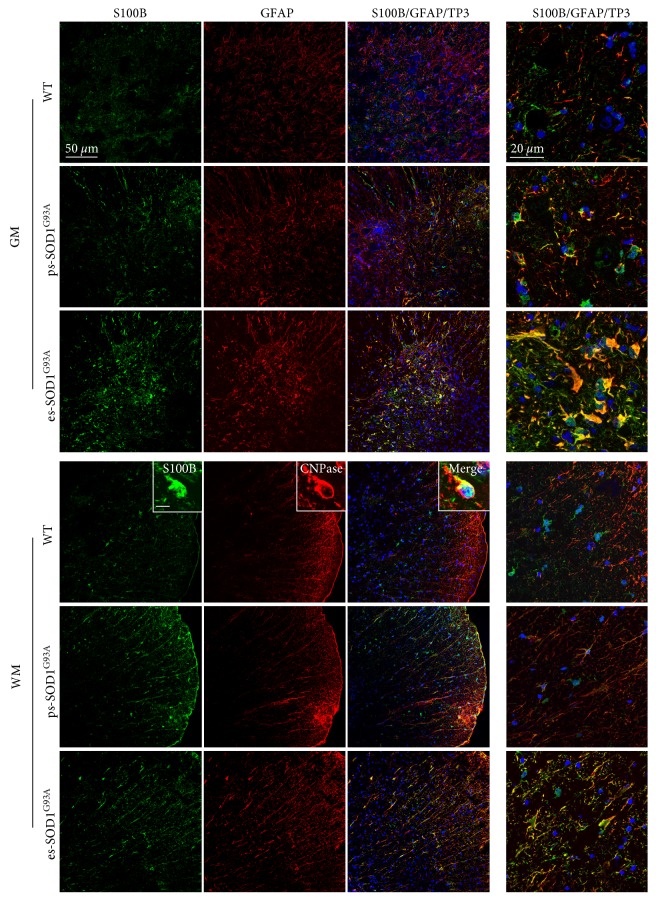
S100B increase in GFAP-positive cells in the lumbar spinal cord from SOD1^G93A^ rats. Double immunofluorescence labelling was performed with anti-S100B (green) and anti-GFAP (red) in the lumbar spinal cord from wild-type (WT), presymptomatic (ps-SOD1^G93A^), and end-stage (es-SOD1^G93A^) rats. The images were acquired from the grey matter (GM) and from the white matter (WM) at two magnifications (scale bars: 50 *μ*m left columns and 20 *μ*m right column). Merged panels also show To-Pro-3 nuclear staining (TP3, blue). In WM from WT rats, insets show a double immunolabelling with anti-S100B (green) and anti-CNPase (red) and merged inset also with To-Pro-3 staining (scale bar: 10 *μ*m).

In the ventral horn of the lumbar spinal cord from WT rats, S100B is barely detectable, while the expression of GFAP is mild, with positive cells exhibiting the canonical morphology of resting astrocytes (Figure 1, WT - GM). In the white matter (WM), S100B and GFAP are poorly overlapping (Figure 1, WT - WM) and S100B is essentially expressed by GFAP-negative roundish cells displaying one or two processes, which are recognized by the mature oligodendrocyte marker CNPase (Figure 1, WT - WM insets). In the lumbar spinal cord from SOD1^G93A^ rats, astrocytes progressively acquire a reactive morphology, with an increase of GFAP staining (Figure 1). In ps-SOD1^G93A^ rats, S100B-positive cells clearly appear, mainly at the rim of the WM and at the boundary region between the ventral horn and the WM and their staining mostly overlaps with the signal provided by GFAP (Figure 1, ps-SOD1^G93A^ - WM and GM). In es-SOD1^G93A^ rats, S100B dramatically increases both in WM and GM, evidencing a typical staining of, respectively, fibrous and protoplasmic astrocytes, with an almost complete overlapping with the signal produced by GFAP (Figure 1, es-SOD1^G93A^ - WM and GM). S100B overexpression was also confirmed in the SOD1^G93A^ mice spinal cord (data not shown). Hence, while in control conditions S100B expression is low and mainly confined to oligodendrocytes in the WM, in ALS rats, S100B gradually increases in GM and WM, in GFAP-positive cells. By double labelling for S100B and NeuN or Iba1, we further excluded the possibility that S100B may also be expressed by neurons and microglia in pre-symptomatic or terminal phases of the disease (Supplementary Figure S2A and B).

### 3.2. The Presence of RAGE in the Lumbar Spinal Cord Gradually Shifts from Motor Neurons to GFAP/S100B-Positive Astrocytes during the Course of the Disease

**Figure 2:**
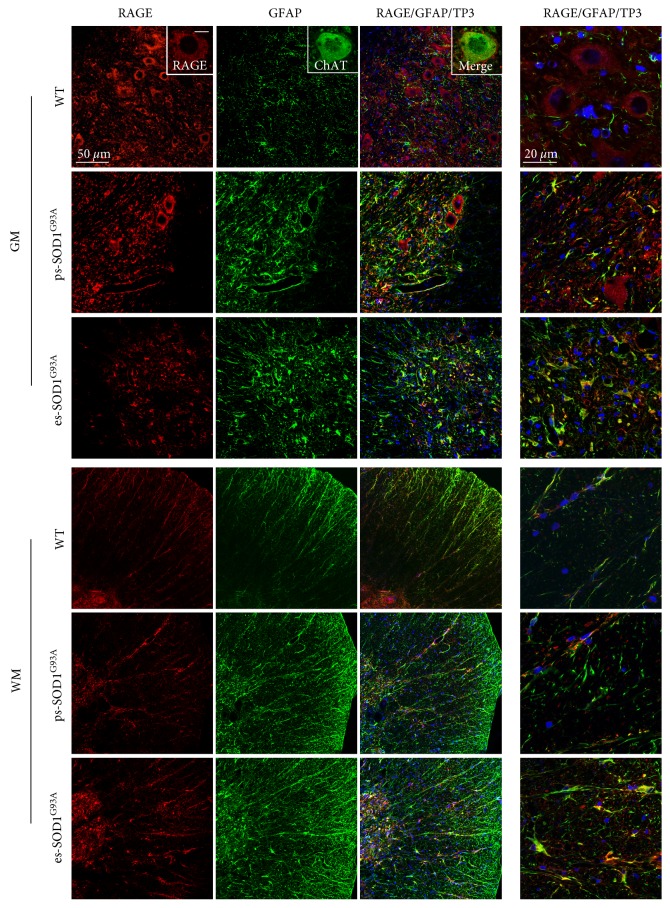
RAGE increase in GFAP-positive cells in the lumbar spinal cord from SOD1^G93A^ rats. Double immunofluorescence was performed with anti-RAGE (red) and anti-GFAP (green) on lumbar spinal cord from wild-type (WT), presymptomatic (ps-SOD1^G93A^), and end-stage (es-SOD1^G93A^) rats. The images were acquired from grey (GM) and white matter (WM) at two magnifications (scale bars: 50 *μ*m left column and 20 *μ*m right column). Merged panels also show To-Pro-3 nuclear staining (TP3, blue). In GM from WT rats, insets show a double immunofluorescence with anti-RAGE (red) and anti-ChAT (green) (scale bar: 10 *μ*m).

We then investigated the expression and localization of the S100B receptor/effector RAGE during the course of the disease, with the use of an antibody raised against the N-terminal of the protein and thus able to recognize both the FL and the soluble forms of the receptor. The use of the C-terminal-directed anti-RAGE antibody, which recognizes essentially the FL receptor, was not possible for immunofluorescence purposes as it provides an unspecific nuclear staining (data not shown). We performed this analysis first assessing the presence of RAGE in astrocytes in the lumbar spinal cord. In WT rats, RAGE shows an evident neuronal localization and marks motor neurons in the ventral horn region, as indicated by coexpression with the typical motoneuronal marker ChAT (Figure 2, WT - GM insets), with no overlapping signal with GFAP (Figure 2, WT - GM). In ps-SOD1^G93A^ rats, there is a loss of RAGE-positive motor neurons, likely reflecting the decrease of motor neuron number (Supplementary Figure S1A and B), but, interestingly, RAGE appears in GFAP-positive cells (Figure 2, ps-SOD1^G93A^ - GM). This situation is exacerbated in es-SOD1^G93A^ rats where a neuronal RAGE staining is no longer evident, rather, RAGE clearly stains GFAP-positive reactive astrocytes (Figure 2, es-SOD1^G93A^ - GM). In the WM from WT rats, RAGE presence is mild and overlaps with GFAP staining within fibrous astrocytes (Figure 2, WT - WM). In ps- and es-SOD1^G93A^ rats, the staining of RAGE-positive cells in the WM gradually intensifies, along with the increased signal provided by GFAP, showing at the end-stage an almost complete coexpression of the two signals (Figure 2, WM). From these data, it appears that RAGE in control conditions is essentially expressed by motor neurons in the GM and mildly by astrocytes in the WM, but, during the disease, RAGE levels are highly increased in reactive astrocytes both in GM and WM.

**Figure 3:**
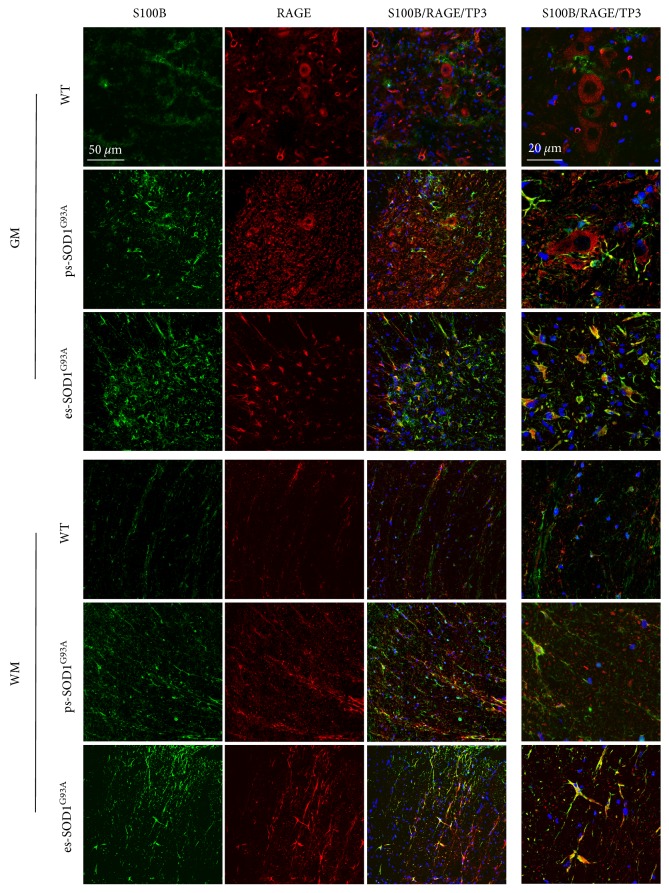
S100B and RAGE colocalization in the lumbar spinal cord from SOD1^G93A^ rats. Double immunofluorescence was performed with anti-S100B (green) and anti-RAGE (red) in lumbar spinal cord from wild-type (WT), presymptomatic (ps-SOD1^G93A^), and end-stage (es-SOD1^G93A^) rats. The images were acquired from grey (GM) and white matter (WM) at two magnifications (scale bars: 50 *μ*m left columns and 20 *μ*m right column). Merged panels also show To-Pro-3 nuclear staining (TP3, blue).

From the previous results, it emerges that both S100B and RAGE, expressed at low levels in astrocytes in control conditions, are increasingly expressed in GFAP-positive cells in presymptomatic and end-stage rats. In order to analyze whether they are overexpressed by the same cells, a double immunofluorescence labelling was performed (Figure 3). In the ventral horn from ps-SOD1^G93A^ rats, RAGE maintains a neuronal expression but also appears in S100B-positive cells, while in the WM RAGE signal almost completely colocalizes with S100B (Figure 3, ps-SOD1^G93A^ - GM and WM). In the es-SOD1^G93A^ rats, where there is a strong increase of S100B-expressing cells and an evident motoneuronal degeneration, RAGE is almost totally localized in the same cells expressing S100B, both in GM and WM (Figure 3, es-SOD1^G93A^ - GM and WM), suggesting therefore the occurrence of an autocrine pathway of S100B in astrocytes.

### 3.3. In the Cervical Spinal Cord, S100B Levels Gradually Increase in Astrocytes While RAGE Presence Shifts from Motor Neurons to GFAP/S100B-Positive Astrocytes Only at End-Stage of the Disease

**Figure 4:**
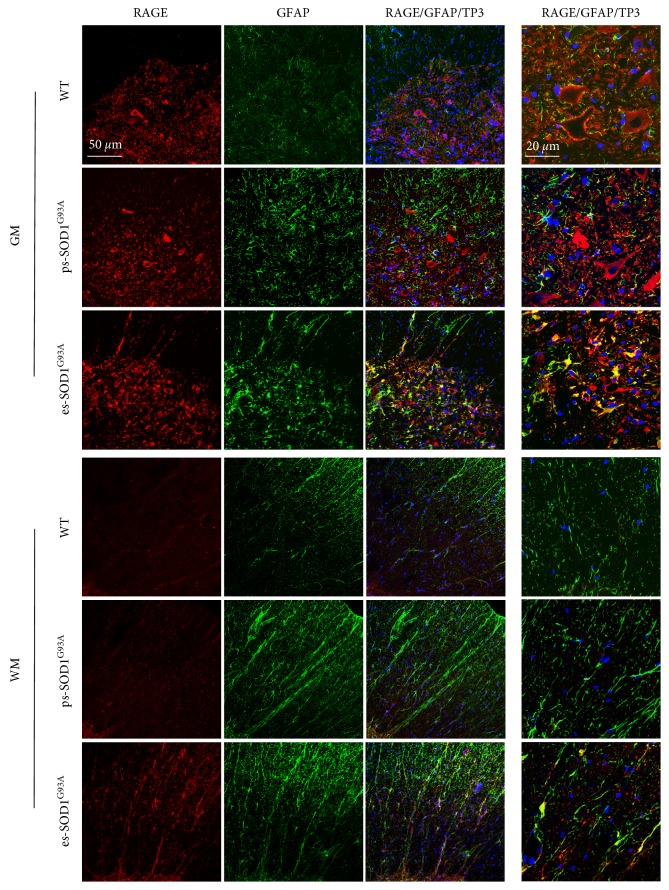
RAGE increase in GFAP-positive cells in the cervical spinal cord from SOD1^G93A^ end-stage rats. Double immunofluorescence was performed with anti-RAGE (red) and anti-S100B (green) in cervical spinal cord from wild-type (WT), presymptomatic (ps-SOD1^G93A^), and end-stage (es-SOD1^G93A^) rats. The images were acquired from grey (GM) and white matter (WM) at two magnifications (scale bars: 50 *μ*m left columns and 20 *μ*m right column). Merged panels also show To-Pro-3 nuclear staining (TP3, blue).

Since we assessed that in our presymptomatic animals the levels of motor neuron degeneration in the cervical spinal cord is negligible (Supplementary Figure S1C), we asked whether also in these conditions of mild impairment, the expression and localization of S100B and RAGE were altered. As shown in Supplementary Figure S3, and in a comparable way to the lumbar spinal cord, S100B-positive cells are barely detectable in WT rats and gradually increase in their number in presymptomatic and end-stage animals. Also in this case, the levels of S100B increase along with GFAP staining and progressively overlap with GFAP-positive reactive astrocytes. As for lumbar spinal tissues, in the ventral horn of the cervical spinal cord from WT animals, RAGE stains motoneuronal cells, without overlapping with GFAP (Figure 4, WT - GM). Nevertheless, differently from lumbar tissues, in ps-SOD1^G93A^ cervical sections, RAGE holds an exclusive neuronal staining, with no evident expression by GFAP-positive cells, although these have started to display a reactive morphology (Figure 4, ps-SOD1^G93A^ - GM and WM). We find RAGE in GFAP-positive cells only in es-SOD1^G93A^ tissues, thus only when motor neuron degeneration becomes evident (Figure 4, es-SOD1^G93A^ - GM). In WM, RAGE signal remains barely detectable until the end-stage condition where it is highly expressed and almost completely overlaps with GFAP-positive cells (Figure 4, WM). We finally investigated also the coexpression of S100B and RAGE demonstrating that only in the es-SOD1^G93A^ rats, where there is an evident motoneuronal degeneration, RAGE colocalizes with S100B-positive cells, both in GM and WM (Supplementary Figure S4).

### 3.4. In SOD1^G93A^ Rats, FL-RAGE Protein Levels and the mRNA Ratio FL-RAGE/sRAGE Vary in the Different Stages of the Disease and Display a Different Pattern of Expression in the Lumbar and Cervical Portions of the Spinal Cord

**Figure 5:**
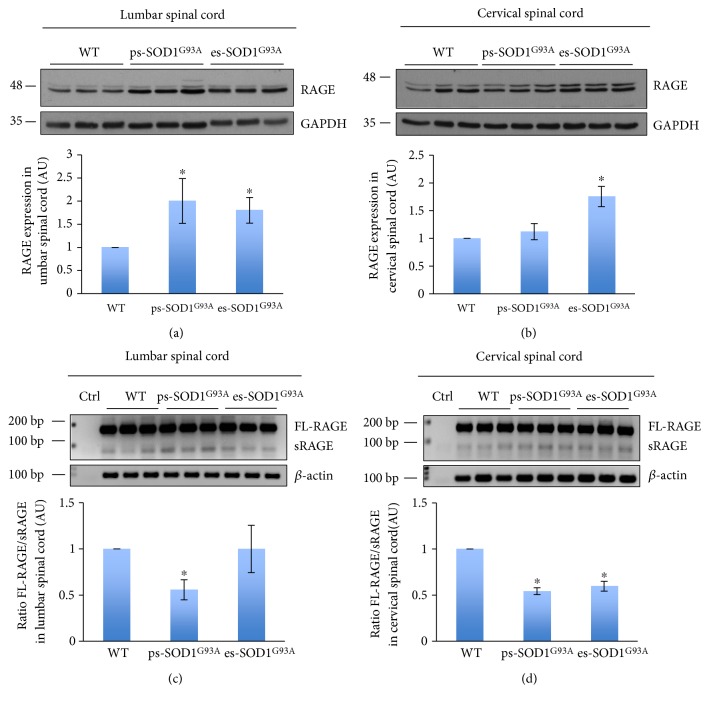
FL-RAGE protein levels and FL/sRAGE mRNAs ratio alterations in spinal cord tissues from SOD1^G93A^ rats. Protein lysates (a, b) and mRNAs (c, d) of lumbar (a, c) and cervical (b, d) spinal cord from wild-type (WT), presymptomatic (ps-SOD1^G93A^), and end-stage (es-SOD1^G93A^) rats were analyzed, respectively, by Western blot (a, b) and semiquantitative PCR (c, d). Anti-RAGE antibody was used to detect the FL-RAGE in the tissues. Anti-GAPDH antibody was used to normalize the samples (a, b). The lower panels of (a) and (b) show signal quantification expressed in arbitrary units (AU), relative to WT animals, and reported as mean ± s.d. (*n* = 3 animals per group). ^∗^*P* < 0.05. FL-RAGE, sRAGE and *β*-actin PCR products are shown (c, d). The lower panels of (c) and (d) show the FL/sRAGE ratio obtained from signal quantification, expressed in arbitrary units (AU) relative to WT animals, and reported as mean ± s.d. (*n* = 3 animals per group). ^∗^*P* < 0.05.

As shown before, both in lumbar and in cervical healthy spinal cord, the localization of RAGE is prevalently neuronal, but, concomitantly with motor neuron loss, it shifts its expression in astrocytes. In order to analyze whether this event is correlated with an expression change of specific RAGE isoforms, we evaluated by Western blot the amount of FL-RAGE, employing an anti-C-terminal RAGE antibody. In the lumbar spinal cord, the levels of FL-RAGE increase of about 2-fold in both presymptomatic and end-stage rats, with respect to control levels (Figure 5(a)), thus reinforcing the immunofluorescence data showing an early (Figure 2) presymptomatic aberrant expression of RAGE. In cervical tissue, FL-RAGE level remains constant in ps-SOD1^G93A^ rats while it displays a 1.75-fold increase in es-SOD1^G93A^ rats (Figure 5(b)). Thus, in parallel with the immunofluorescence data (Figure 4), in the cervical spinal cord, RAGE displays an aberrant expression only at the end-stage of the disease, when most motor neurons have been lost. These data would thus indicate that the FL form of RAGE is increased when motor neuron death becomes evident and when RAGE is mainly expressed by GFAP/S100B-positive astrocytes. We then performed a PCR analysis on lumbar and cervical tissues with a pair of oligonucleotides designed to discriminate between the FL and the s-RAGE isoforms, basing on their length. In the lumbar spinal cord, the ratio between the FL- and s-RAGE decreases by almost 40% in presymptomatic animals and returns to control levels at the end-stage (Figure 5(c)), indicating that in the presymptomatic condition there is an increase in the expression of the soluble form of the receptor. A similar extent of FL-RAGE/s-RAGE ratio decrease (about 40%) is found also in the cervical spinal cord of both ps- and es-SOD1^G93A^ rats (Figure 5(d)), indicating that in the cervical tissue the increase in alternative splicing of producing sRAGE mRNA is maintained up to the end-stage of the disease.

### 3.5. The Mere Presence of SOD1^G93A^ Is Able to Increase the Intracellular Levels and Release of S100B from Astrocytes, Sparing RAGE Protein Expression

**Figure 6:**
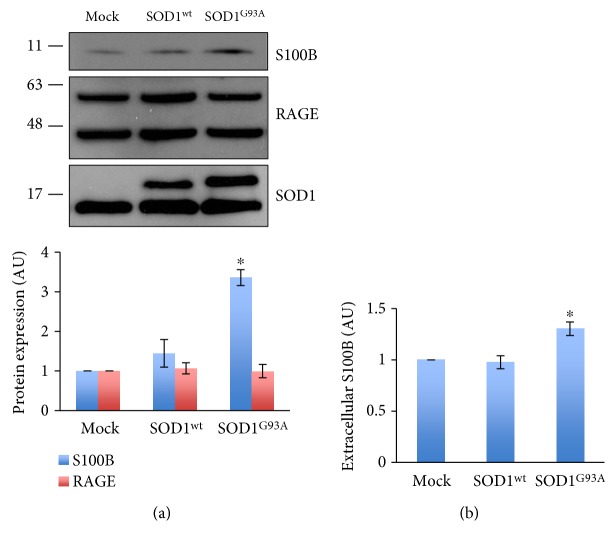
Intracellular and extracellular increases of S100B by transfection of SOD1^G93A^ in C6 cells. C6 cells were transiently transfected with pCMV (mock), pCMV-SOD1^wild-type^ (SOD1^wt^), or pCMV-SOD1^G93A^ (SOD1^G93A^). (a) Cells were lysed and analyzed by Western blotting with anti-S100B, anti-RAGE, and anti-SOD1. The lower panel shows signal quantifications expressed in arbitrary units (AU), relative to mock, and reported as mean ± s.d. (*n* = 3 independent experiments). ^∗^*P* < 0.05. (b) ELISA assay for S100B contained in the supernatant of C6 cells transfected with mock, SOD1^wt^, or SOD1^G93A^ plasmids. Mean ± s.d. (*n* = 3 independent experiments). ^∗^*P* < 0.05.

The analysis of affected tissues from SOD1^G93A^ rats evidenced the overexpression of S100B and RAGE in astrocytes in the progression of the disease, with an extent that is tissue-specific. Aiming to verify whether the alteration of S100B and RAGE could depend on the mere presence of the ALS-linked SOD1^G93A^ protein in astrocytes, we transiently overexpressed human SOD1^G93A^ in C6 glioma cells. By the use of these cells, we could exclude preconditioniong effects which might instead occur to primary astrocytes as a resultant of interactions with surrounding cells and that could affect also the levels of S100B and RAGE. As shown in Figure 6(a), S100B levels were increased by 3 times in C6 cells overexpressing the SOD1^G93A^ gene with respect to mock and SOD1^wt^ controls. In contrast, RAGE levels, analyzed with an N-terminal-directed antibody, were not changed. We further investigated whether the transient overexpression of SOD1^G93A^ was also able to induce S100B release. The ELISA assay performed on C6 cell supernatants shows that the mere presence of SOD1^G93A^ significantly increases S100B release compared to both controls, mock and SOD1^wt^ transfected cells, (Figure 6(b)) without altering cell viability (data not shown).

### 3.6. The Decrease of S100B in SOD1^G93A^ Astrocytes Downregulates the Expression of Proinflammatory Genes

**Figure 7:**
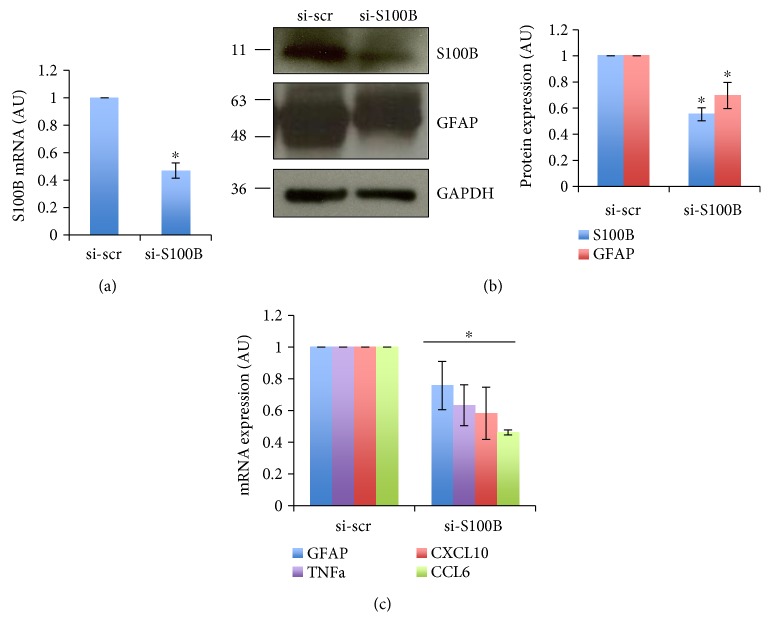
Reduction of proinflammatory genes expression by S100B silencing in SOD1^G93A^ primary astrocytes. Primary astrocytes were transfected with scramble (si-scr) or anti-S100B siRNAs (si-S100B) at 24 and 48 h after plating. 72 hours after plating, cells were lysed, and RNA (a, c) or protein was extracted (b). (a, c) cDNA from si-scr and si-S100B-treated cells was analyzed by real-time qPCR for S100B, GFAP, TNF*α*, CXCL10, and CCL6 expression. The panels show the quantification of each mRNA expressed in arbitrary units (AU) and reported as mean ± s.d., relative to corresponding si-scr (*n* = 3 independent experiments). ^∗^*P* < 0.05. (b) Western blot analysis with anti-S100B, anti-GFAP, and anti-GAPDH. In the right panels, the quantification of S100B (blue) and GFAP (red) bands normalized to GAPDH and relative to si-scr. Data are expressed as mean ± s.d. (*n* = 3 independent experiments). ^∗^*P* < 0.05.

From the previous results, a relationship emerges between the intracellular and extracellular increases of S100B and the overexpression of a mutant SOD1 linked to ALS. Since primary astrocytes from rodent pups are known to display a reactive neurotoxic phenotype, we finally investigated if the inhibition of S100B expression by RNAi in astrocytes is sufficient to reduce several reactivity markers. To this purpose, we transiently transfected primary astrocytes derived from mouse pup cortices with a scrambled siRNA (si-scr) and a siRNA-targeting S100B (si-S100B). In si-S100B-treated astrocytes, S100B mRNA decreases by 55%, compared to astrocytes transfected with the scrambled siRNA (Figure 7(a)) and, likewise, the concentration of S100B protein decreases by 45% with respect to control (Figure 7(b)). We then evaluated the effect of S100B silencing on the proinflammatory signature of SOD1^G93A^ astrocytes, by analyzing the mRNA level of several genes found to be increased in ALS astrocytes, that is, GFAP, TNF-*α*, CXCL10, and CCL6. The reduction of S100B in SOD1^G93A^ astrocytes decreases the expression of all of them: GFAP by 25%, TNF by 35%, CXCL10 by 40%, and CCL6 by 55% (Figure 7(c)). We also verified a 30% decrease in expression of GFAP protein in si-S100B-transfected astrocytes (Figure 7(b)). Overall, these data would indicate that S100B may influence the expression of a proinflammatory phenotype in mutant SOD1 astrocytes.

## 4. Discussion

Over the last decade, RAGE has gained attention as a regulator of innate and adaptive immunity in different pathologies associated with neuroinflammation, such as AD, PD, Huntington's disease (HD), Creutzfeldt-Jacob disease (CJD), diabetic neuropathy, and Charcot neuroartropathy (for review [34]). While the specific molecular mechanism by which RAGE contributes to neurodegeneration remains elusive, studies indicate that the detrimental actions of RAGE, in the absence of a pathogen, are triggered upon its binding to certain ligands, collectively called DAMPs, among which is S100B. With this study, we illustrate a clear dysregulation and a possible proinflammatory role for the S100B-RAGE pathway in ALS, another neurodegenerative disease characterized by a strong neuroinflammatory component. So far, only partial and descriptive studies were available, essentially evidencing the overexpression of S100B and RAGE. By the immunofluorescence analysis performed in both lumbar and cervical spinal cord of SOD1^G93A^ rats, we provided a detailed description of the localization of both S100B and RAGE at two stages of the disease, presymptomatic and terminal. In particular, we have shown that S100B is expressed at low levels in healthy animals, as it was expected. Its localization is mostly restricted to the white matter, where it also appears in CNPase-positive mature oligodendrocytes, which staining instead decreases in affected tissues probably due to oligodendrocyte degeneration, a recently described phenomenon occurring in both patients and rodent models of the disease [35]. Overall, S100B staining increases in SOD1^G93A^ tissues, and, in particular, it is massively expressed by GFAP-positive astrocytes with typical reactive phenotypes. It was previously reported that ALS patients possess increased immunoreactivity for S100B protein in brain and spinal cord, but with different findings about its localization. It was found either in clustered astrocytes in the primary motor cortex [21], or both in astrocytes and motor neurons from patients' spinal cord [22]. A later report obtained with a rat model of the disease identified S100B in a subpopulation of GFAP-positive astrocytes defined as “aberrant,” labelled with the proliferation marker Ki67 and characterized by increased toxic properties towards motor neurons [26]. Ki67-positive cells were also shown to display the coexpression of GFAP and microglial markers in symptomatic rats, and in vitro experiments demonstrated that they might derive from the phenotypic transition of phagocytic microglial cells into an astrocytic population [36]. Here, we demonstrated that S100B does not colocalize with NeuN, nor with Iba1 in both analyzed stages of the disease, confining its expression to astrocytes. Therefore, the previously reported S100B presence in motor neurons [22] may be restricted to the human samples and represent a peculiar localization pattern. Furthermore, we have also ruled out, at least in the presymptomatic and terminal stages of the disease, that S100B-positive astrocytes may possess microglial features and thus that in vivo they may derive from microglial cells. The astrocytic-restricted localization of S100B during the progression of the disease is consistent with the expression pattern of the molecule in other neurodegenerative diseases, such as HD [37] and PD [20], but differs from AD where, in the double-transgenic PS/APP model, S100B also stains Iba1-positive cells [38]. In addition, we have demonstrated that the increase of S100B in astrocytes occurs not only when an overt motoneuronal damage takes place (as at the presymptomatic stage in the lumbar spinal cord or at end-stage situations), but also when motor neuron death is not apparent, as in the presymptomatic cervical segment of the spinal cord. In this context, astrocytes display a moderate reactive phenotype and their response may involve either a supportive effect or a neurotoxic secretion of proinflammatory factors [39]. In any case, S100B behaves as a presymptomatic marker of astrocytic reactivity in vivo. Under this regard, although ALS is a multifactorial, multisystemic disease with a strong inflammatory component [40], one of the main questions regarding the activation of glial cells is whether this is a secondary effect due to massive motor neuron death or glial cells might develop an active phenotype also independently of signaling from dying motor neurons. There are many observations showing that glial cell activation occurs prior to an evident motor neuron death [41, 42], and, in this case, inflammation might contribute actively also to the first stages of the disease and possibly to the initial motor neuron impairment. We have shown here that the mere transfection specifically of the SOD1^G93A^ gene in C6 glioma cells is sufficient to elicit an upregulation of S100B production and release. In this way, we have demonstrated that there is a direct relationship between mutant SOD1 presence in astrocytes and S100B dysregulation, and this would suggest, with the limitations of the experimental model in mind, that during the course of the disease S100B upregulation and release might be early events taking place in astrocytes (as also indicated by the immunofluorescence data on cervical presymptomatic tissues) that could occur also independently of motor neuron death. This relationship could be explained by the capability of mutant SOD1 to alter the concentration of calcium in astrocytes [43], and by the fact that S100B is a calcium-binding protein, the stability and secretion of which can be controlled by intracellular calcium levels [44]. Since the presence of a mutant form of SOD1 induces also the release of S100B from astrocytes, we may speculate that S100B in ALS could behave as a proinflammatory factor, capable of affecting motor neuron viability. This hypothesis is also in line with the observation of the increased neurotoxic potential displayed by the conditioned medium derived from S100B highly expressing “aberrant” astrocytes [26] that may contain S100B among other harmful soluble molecules.

In support of an extracellular role for S100B in ALS, we have also reported a dysregulation of RAGE expression levels and distribution. The receptor is present essentially in neurons (including motor neurons) in control conditions, while during the progression of the disease it shifts its localization to astrocytes. While the loss of motor neuron staining is readily explained by increased motor neuron death that characterizes the course of ALS, the appearance of the signal in astrocytes may instead contribute to the pathogenic mechanism of the disease and, interestingly, was also observed in spinal cord from ALS patients [27]. Immunofluorescence analysis obtained using an antibody incapable of discriminating FL- from s-RAGE isoforms shows that RAGE increases in astrocytes in the presymptomatic stage only in the lumbar spinal cord. Moreover, the Western blot analysis performed using an antibody that recognizes essentially the FL-RAGE protein shows that this isoform displays an expression pattern that is in line with the immunofluorescence data: it is increased already in the presymptomatic stage in the lumbar spinal cord, while it is upregulated only at the end stage in the cervical segment. These data would suggest that FL-RAGE might increase in astrocytes when motor neuron death becomes significant, consistently with the possibility that an autocrine loop of RAGE activation in astrocytes, dependent on signals released by dying motor neurons, may occur. Actually, the absence of alteration of RAGE levels in SOD1^G93A^-transfected C6 cells in culture is in line with this hypothesis. It may also be relevant in this respect that a RAGE-dependent autocrine loop of S100B, turning astrocytes into a reactive phenotype characterized by a proinflammatory-neurodegenerative profile that facilitated death of oxygen/glucose deprivation-exposed neurons, has been demonstrated [17]. Nevertheless, we have also shown that the ratio between the FL- and the soluble form of the receptor decreases in the presymptomatic stage in both lumbar and cervical spinal cord, persisting in this altered situation only in the cervical spinal cord in the terminal stage. We may thus speculate that the ligand-scavenging isoform of RAGE is expressed essentially by residual motor neurons (almost absent in the lumbar spinal cord at end-stage) as a protective mechanism against ligand- (among which S100B) induced death. We may thus hypothesize that when motor neuron impairment becomes consistent, astrocytes produce and respond to S100B upregulating the FL-form of RAGE, while residual motor neurons may increase the levels of s-RAGE in an attempt to protect themselves from toxicity induced by RAGE ligands. Finally, we explored the role of S100B in SOD1^G93A^ astrocytes by RNAi experiments. Since intracellular S100B is regarded to regulate many cellular processes as a result of changes of intracellular Ca^2+^ concentrations (for review see [15]), we analyzed if its inhibition in astrocytes derived from SOD1^G93A^ mice could affect the expression of inflammatory genes that were found heightened in ALS astrocytes. We chose in particular those genes that were found mostly increased specifically in astrocytes in early phases of the disease in the mouse model [45], as well as in human astrocytes (from both familial and sporadic ALS) [10]. We have thus demonstrated that the inhibition of S100B downregulates the expression of GFAP and of the cytokines TNF*α*, CCL6, and CXCL10, indicating that the protein might promote a proinflammatory phenotype in SOD1^G93A^ astrocytes.

## 5. Conclusion

Our data pose S100B as a key player in tuning the inflammatory response of astrocytes and in managing the interplay between astrocytes and surrounding cells. Whether the aberrant dysregulation of S100B and its receptor RAGE are among the causes or the hallmarks of ALS has yet to be elucidated. Nevertheless, our findings propose S100B-RAGE axis as a relevant contributor to the pathogenesis of the disease, and its blockade may be suggested as a rational target for therapeutic intervention in ALS. It is reasonable therefore to speculate that the inhibition of S100B in vivo may reduce the proinflammatory features of astrocytes and influence in this way the progression of the disease.

## Supplementary Material
