# Supplementary figures and images for: The Astrocytic S100B Protein with Its Receptor RAGE Is Aberrantly Expressed in SOD1G93A Models, and Its Inhibition Decreases the Expression of Proinflammatory Genes
Source: Mediators Inflamm. 2017 Jun 20;2017:1626204. doi: 10.1155/2017/1626204 (PMC5496121; doi:10.1155/2017/1626204)

(A)

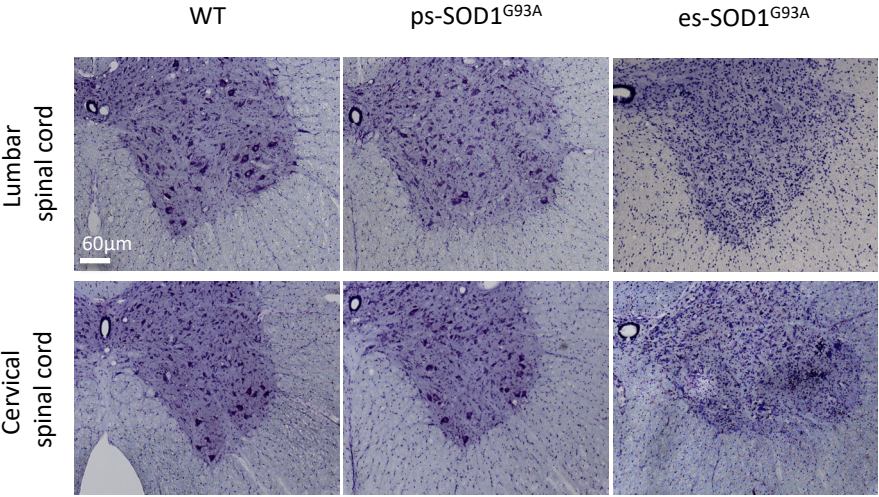

(B)

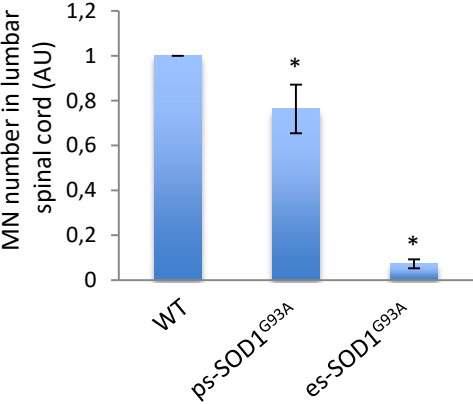

(C)

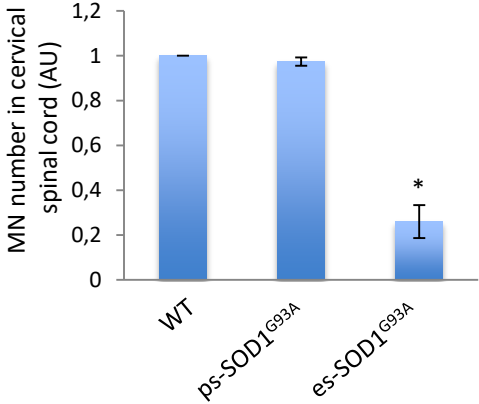

Supplement: Supplementary file 1 [file 1626204.f1.pdf]

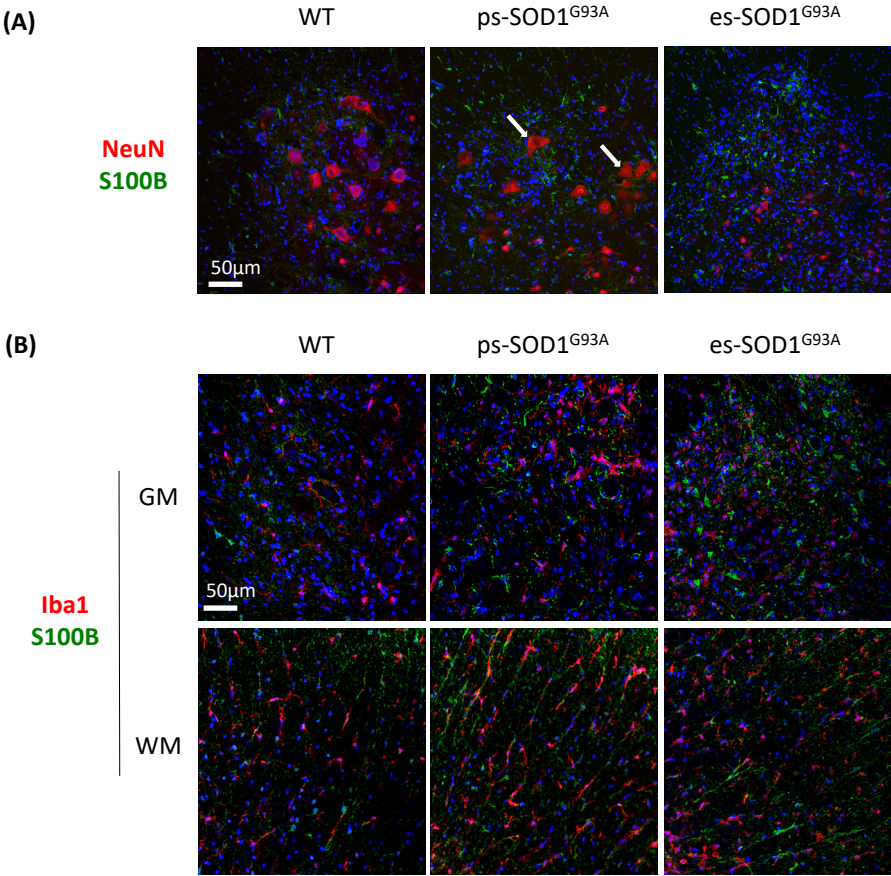

Supplement: Supplementary file 2 [file 1626204.f2.pdf]

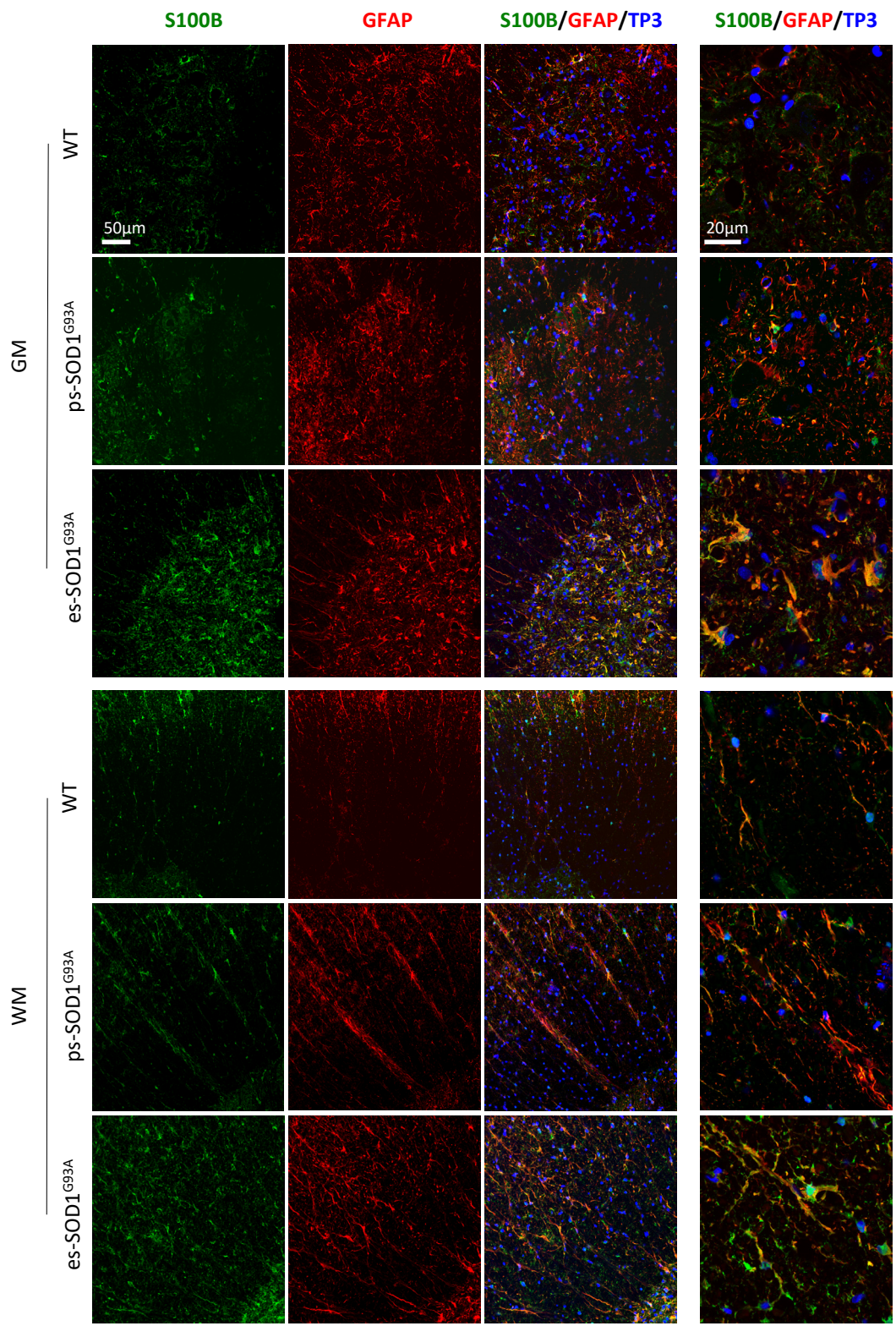

Supplement: Supplementary file 3 [file 1626204.f3.pdf]

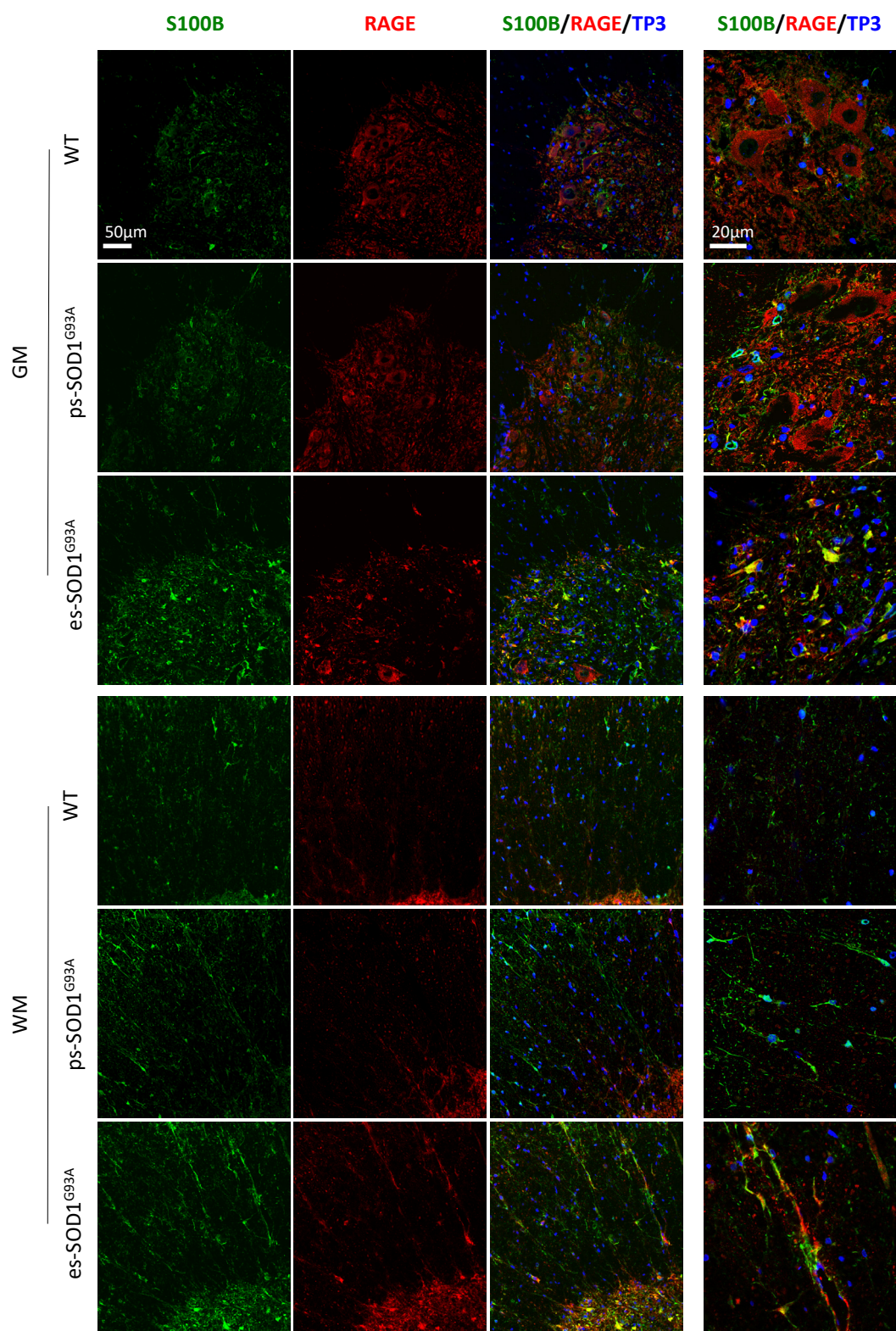

Supplement: Supplementary file 4 [file 1626204.f4.pdf]
